# Postoperative Packing of Perianal Abscess Cavities (PPAC2): randomized clinical trial

**DOI:** 10.1093/bjs/znac225

**Published:** 2022-08-05

**Authors:** Katy Newton, Jo Dumville, Michelle Briggs, Jennifer Law, Julia Martin, Lyndsay Pearce, Cliona Kirwan, Thomas Pinkney, Alexander Needham, Richard Jackson, Simon Winn, Haley McCulloch, James Hill, PPAC2 Collaborators, A Watson, M Johnson, L Hiller, E Psarelli, L Murray, A Smith, S Brown, B Singh, C Newby, O Ali, A Sukha, N Blencowe, S Narang, N Reeves, G Faulkner, S Rajamanickam, J Evans, S Mangam, M Harilingham, C J Smart, S J Ward, M Bogdan, K Amin, Z Al-Khaddar, E Davies, P Patel, A Stearns, I Shaik, J Hernon, A Pal, M Lewis, J Barker, A Gerrard, M Abdel-Halim, P Shuttleworth, M J Lee, A B P Peckham-Cooper, A G Hague, C Challand, C Steele, N Fearnhead, S Van Laarhoven, R Brady, F Shaban, N Wong, W Ngu, G Williams, R Codd, D Magowan, K Leong, G Williams, A Torrance, B Bharathan, N Pawa, H Kaur Sekhon, I Singh, A Alabi, D Berry, V Trompetas, J L Hughes, R Lunevicius, R Lunevicius, K Mann, S Dixon, T Ingram, T Gilbert, C Brooks, G Madzamba, A Pullyblank, G Dovell, L Newton, N Carter, P May-Miller, S Shaikh, R Shearer, C Macleod, C Parnaby, A Abdelmabod, L Titu, T Majeed, R Hargest, J Parker, C Zabkiewicz, N Reeves, F Soliman, G Gossedge, H Selvachandran, M Dilworth, D Vimalachandran, H Singh, H Koh, J Randall, S Moug, A Adeosun, G Dennison, N Curtis, N Smart, S Duff, M Rahman, F Wu

**Affiliations:** Department of General Surgery, Manchester Royal Infirmary, Manchester University NHS Foundation Trust, UK; School of Midwifery, Nursing and Social Work, University of Manchester, UK; School of Midwifery, Nursing and Social Work, University of Manchester, UK; North West Research Collaborative, UK; North West Research Collaborative, UK; Department of General Surgery, Salford Royal NHS Foundation Trust, UK; Department of Academic Surgery, Wythenshawe Hospital, Manchester University NHS Foundation Trust, UK; Academic Department of Surgery, University of Birmingham, UK; Liverpool Clinical Trials Unit, UK; Liverpool Clinical Trials Unit, UK; Liverpool Clinical Trials Unit, UK; Liverpool Clinical Trials Unit, UK; Department of General Surgery, Manchester Royal Infirmary, Manchester University NHS Foundation Trust, UK

## Abstract

**Background:**

Perianal abscess is common. Traditionally, postoperative perianal abscess cavities are managed with internal wound packing, a practice not supported by evidence. The aim of this randomized clinical trial (RCT) was to assess if non-packing is less painful and if it is associated with adverse outcomes.

**Methods:**

The Postoperative Packing of Perianal Abscess Cavities (PPAC2) trial was a multicentre, RCT (two-group parallel design) of adult participants admitted to an NHS hospital for incision and drainage of a primary perianal abscess. Participants were randomized 1:1 (via an online system) to receive continued postoperative wound packing or non-packing. Blinded data were collected via symptom diaries, telephone, and clinics over 6 months. The objective was to determine whether non-packing of perianal abscess cavities is less painful than packing, without an increase in perianal fistula or abscess recurrence. The primary outcome was pain (mean maximum pain score on a 100-point visual analogue scale).

**Results:**

Between February 2018 and March 2020, 433 participants (mean age 42 years) were randomized across 50 sites. Two hundred and thirteen participants allocated to packing reported higher pain scores than 220 allocated to non-packing (38.2 *versus* 28.2, mean difference 9.9; *P* < 0.0001). The occurrence of fistula-in-ano was low in both groups: 32/213 (15 per cent) in the packing group and 24/220 (11 per cent) in the non-packing group (OR 0.69, 95 per cent c.i. 0.39 to 1.22; *P* = 0.20). The proportion of patients with abscess recurrence was also low: 13/223 (6 per cent) in the non-packing group and 7/213 (3 per cent) in the packing group (OR 1.85, 95 per cent c.i. 0.72 to 4.73; *P* = 0.20).

**Conclusion:**

Avoiding abscess cavity packing is less painful without a negative morbidity risk.

**Registration number:**

ISRCTN93273484 (https://www.isrctn.com/ISRCTN93273484).

**Registration number:**

NCT03315169 (http://clinicaltrials.gov)

## Teaser

The PPAC2 randomized clinical trial investigated the role of continued internal wound packing after incision and drainage of acute perianal abscess, a common general surgical condition. Across 50 NHS sites, 433 participants were recruited and randomized to receive packing or no packing, and were followed up for 6 months. No packing was found to be associated with significantly less pain, and no significant increase in fistula-in-ano or abscess recurrence.

## Introduction

Perianal abscess is a common acute general surgical condition^1^. Ninety per cent are cryptoglandular in origin and caused by blockage and infection of the anal glands within the intersphincteric space of the anal canal^2,3^. Inflammatory bowel disease is a further important cause. Infection can spread downwards to the perianal skin, alongside the rectum (ischiorectal), above the pelvic floor (supralevator), or between the internal and external sphincters (intersphincteric)^4^. Other risk factors include smoking (twofold risk) and diabetes mellitus. Perianal abscess is twice as common in men as women, and the mean age at presentation is 40 years^1,5,6^.

The management of acute perianal sepsis has remained largely unchanged for over 50 years. Surgical abscess incision and drainage under general anaesthesia is followed by continued internal wound dressing (packing) of the resultant wound cavity in the community until the wound has healed. Pack changes are required multiple times a week for several weeks^7^. Packing is an accepted treatment^7^. Surgical dogma is that packing reduces the rate of perianal fistula formation and abscess recurrence, but there is no evidence to support this. Perianal fistulae (epithelial-lined tract between the perianal skin and anal canal or rectum) are sequelae of perianal abscesses, causing pain, purulent discharge, and almost invariably require surgical treatment, which may be complex. The majority of patients presenting with a fistula have a clear history of a previous abscess^8^. Fistula incidence after perianal abscess is variably reported; 5 to 37 per cent from small case series^3,9^, 27 per cent from our pilot data^7^, and 15.5 per cent from a large population-based study^1^. There is limited specific guidance on the postoperative management of the abscess cavity in existing national guidelines^10,11^. To date, two underpowered trials with only 64 participants investigating the role of packing in this setting have been reported^12,13^.

This group previously reported a pilot observational and feasibility study of 141 patients^7^. Results showed that wound packing is painful with a two- to three-fold increase in patient reported pain scores (visual analogue score (VAS)) during and after dressing changes^7^. On average (mean), each patient underwent 13 pack changes in the first 3 weeks of treatment (range 0 to 21). Patient-reported outcomes are an increasingly important measure of the quality of surgical care. Pain has been found to significantly impact on overall patient experience. Notably, moderate or severe postoperative pain, which occurs more frequently than a postoperative complication, has been shown to be strongly associated with a lower likelihood of the patient being satisfied after surgery. A patient with severe pain is nearly twice as likely to be unsatisfied or regret surgery than a patient who experienced a complication^14^. The objective of the Postoperative Packing of Perianal Abscess Cavities (PPAC2) trial was to determine whether non-packing of perianal abscess cavities is less painful than packing, without an increase in perianal fistula and abscess recurrence.

## Methods

### Study design and participants

PPAC2 was a multicentre randomized clinical trial (standard two-group parallel design) performed across 50 NHS Acute Hospital Trusts in England, Wales, and Scotland between February 2018 and February 2020. Patients aged 18 years or over presenting with their first perianal abscess of cryptoglandular origin requiring an incision and drainage operation were eligible for inclusion. Exclusion criteria were perianal fistula, Fournier’s gangrene, and horseshoe abscess. All potential participants received an information leaflet. The trial was approved by Greater Manchester West Research Ethics committee (17/NW/0529). The trial was pre-registered with ClinicalTrials.gov in October 2017 (ClinicalTrials.gov Identifier: NCT03315169) with an analysis plan.

### Randomization and masking

Participants were randomized postoperatively prior to hospital discharge to reduce bias through modification of the operative procedure (preoperative recruitment was allowed). Participants were randomly allocated on a 1:1 basis (using the participating site as a stratification factor), via the online Treatment Allocation RanDomIsation System (TARDIS) to receive current standard wound care with internal packing by nurses in the community (control packing arm) or to receive no packing (simple external dressings only). Participants were recruited and randomized by surgeons (trainees and consultants) and research nurses.

It was not feasible to blind the participants, site enrolment personnel, or community nurses as different information, dressings, and patient advice were given to the packing and no-packing groups. The clinical assessor (consultant or appropriately trained trainee surgeon) of wound healing, perianal fistula, and abscess recurrence remained blind to study allocation. The wound dressing was removed by a different member of the research team to ensure blinding of the assessor.

### Procedures

All participants had standard intraoperative wound packing (for haemostasis), removed within the first postoperative 24 hours. In the packing group, the frequency, type of packing dressing, and duration of packing was left to the discretion of the treating nurses, as is current standard practice. In the no-packing group, the use of external absorbent dressings or pads was again left to the discretion of the nurses and the participants, in keeping with the pragmatic design.

Baseline data, including demographics, operative data, health-related quality of life (HRQoL) score (EQ-5D^TM^; EuroQoL Group, Rotterdam, the Netherlands), and postoperative metronidazole use, were recorded. Participants were discharged home with a symptom diary. Daily acute pain assessments (0–100 mm point VAS) of worst daily pain (where 0 is no pain and 100 is worst pain imaginable), and pain before, during, and after dressing changes (for up to 21 days) were recorded. HRQoL score, return to work, participant satisfaction with wound management (5-point scale of strongly disagree, disagree, neutral, agree, strongly agree), and participant global assessments (3-point scale of poor, fair, and good) were also recorded. Participants were contacted by telephone on day seven to record return-to-work data and to encourage data entry. Participants were reviewed in clinic at 4, 8, and 26 weeks to assess for fistula, abscess recurrence, and wound healing. Wound healing was defined as complete epithelialization of the wound. The 8-week appointment was replaced with a telephone call if the wound had healed at 4 weeks. Fistula and abscess recurrence were serious adverse events (SAEs) and were reviewed and reported even when patients were lost to standard trial follow-up.

### Outcomes

The primary outcome was mean maximum pain score over the first 10 days after incision and drainage surgery (mean of worst daily wound-related pain scores measured on a 100 mm VAS where 0 is no pain, and 100 is worst pain imaginable). Pain was selected as the primary outcome measure for this trial after it was highlighted as the key outcome for patients during patient and public involvement (focus groups) in the PPAC feasibility study^7^. Secondary outcomes included pain before, during, and after dressing changes; postoperative perianal fistula and abscess recurrence (observed during a clinic appointment or recorded as a SAE); bleeding (requiring transfusion or return to theatre); and rate of wound healing (at 4, 8, and 26 weeks). Further patient-reported secondary endpoints included HRQoL (EQ-5D), return to work, participant satisfaction with wound care, and participant global assessment of care.

### Sample size

Sample size calculation was based on the primary outcome mean maximum pain intensity score over the first 10 days after surgery. Pilot data from the PPAC study demonstrated a mean (s.d.) maximum pain intensity score over days 1 to 5 of 40 (27) mm (VAS). Considering a clinically relevant difference in mean maximum pain intensity to be a reduction in severity by 20 per cent^15,16^ (an absolute reduction of 8 mm) and using a two-sided alpha level of 0.05, a sample size of 526 patients (263 in each arm) was required to obtain 90 per cent power (inclusive of a 10 per cent dropout rate). The sample size calculation was adjusted at interim analysis in November 2019. Standard deviation from trial data was less than predicted at 21.5 mm. Sample size was recalculated using this reduction from the assumed s.d. and an updated dropout rate. A sample size of 432 patients was required to provide 85 per cent power to detect a difference in mean maximum pain score of 20 per cent (allowing for the reported missing primary outcome data).

### Statistical analysis

Statistical analysis was performed by the North West Surgical Trials Centre. Continuous data were summarized as means (s.d.) and medians (i.q.r.). Categorical data were assessed as frequency counts and associated percentages. Analyses were performed on an intention-to-treat principle retaining all participants in their randomized groups, irrespective of any protocol violations. A number of sensitivity analyses including a per-protocol analysis were conducted. The primary outcome was defined as the mean maximum pain score over 10 days following the incision and drainage surgery. Only participants who returned at least three pain measurements were included in the analysis. Missing data were handled by investigating the pattern of missingness between treatment groups and other patient characteristics. Multivariable logistic regression was performed to identify factors associated with missing primary endpoint data. The analysis of the primary outcome between groups was performed using a Wilcox rank-sum test. A *P* value of 0.05 was used to determine statistical significance. All analyses were performed using Stata (version 15; StataCorp, College Station, TX, USA).

## Results

**Fig. 1:**
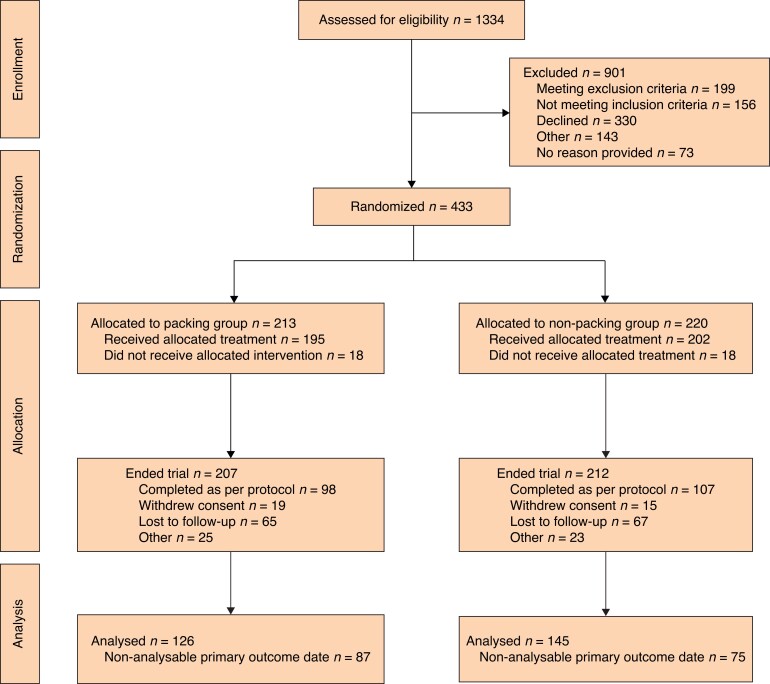
CONSORT diagram

In total, 433 participants were recruited between 1 February 2018 and 1 February 2020 across 50 acute hospital NHS trusts in the UK. Of those randomized, 213/433 (49 per cent) were allocated to packing, and 220/433 (51 per cent) to no packing. Eighteen (8.0 per cent) in the packing group and 18 (8.1 per cent) in the no-packing group did not receive the allocated intervention (*Fig. 1*). The reasons for treatment arm crossover (crossover from packing to non-packing) were as follows: five found packing too painful; the cavity was too small to pack in three; three patients chose not to receive packing; three were crossed over in error; and four participants received a combination of packing and no packing. The reasons for crossover from no packing to packing are as follows: three participants chose to pack for fluid control/toileting, four made a medical decision to pack; eight participants chose to pack; and three were crossed over in error.

Twenty-seven randomized participants were then found to not meet the inclusion criteria and therefore did not proceed in the trial (11/213 (5.0 per cent) of the packing group and 16/220 (7.3 per cent) of the no-packing group). There were 17 other major protocol deviations (9/213 in the packing group and 8/220 in the non-packing group). Follow-up was completed in June 2020, 6 months after the last participant was recruited. Baseline questionnaires and operative data were available for all participants. Baseline demographics were well matched (*Table 1*).

**Table 1:** Patient demographics

Variable	Overall (*n* = 433)	Packing group (*n* = 213)	No-packing group (*n* = 220)
**Median (i.q.r.) age (years)**	42 (31–52)	42 (14)	42 (15)
**Median (i.q.r.) height (cm)**	173 (166–180)	172 (11)	174 (10)
**Median (i.q.r.) weight (kg)**	85 (70–96)	84 (23)	86 (21)
**Smoking status**			
Never	150 (35)	75 (35)	75 (34)
Current smoker	178 (41)	91 (43)	87 (40)
Ex-smoker	97 (22)	42 (20)	55 (25)
Missing	8 (2)	5 (2)	3 (1)
**Sex**			
Female	144 (33)	77 (36)	67 (30)
Male	289 (67)	136 (64)	153 (70)
Missing	0 (0)	0 (0)	0 (0)
**Diabetes**			
No	359 (83)	177 (83)	182 (83)
Yes	62 (14)	28 (13)	34 (15)
Missing	12 (3)	8 (4)	4 (2)
**Anticoagulants or immunosuppressants**			
No	389 (90)	186 (87)	203 (92)
Yes	39 (9)	22 (10)	17 (8)
Missing	5 (1)	5 (2)	0 (0)

In total, 98/213 (46 per cent) participants in the packing group and 107/220 (49 per cent) in the non-packing group completed the trial as per protocol. A similar loss to follow-up rate occurred in both arms (packing group: 65/207 (31 per cent); no-packing group: 67/220 (30 per cent)).

### Missing data

Missing primary outcome data occurred for 87/213 (41 per cent) patients in the packing group and for 75/220 (34 per cent) in the no-packing group (162 patients in total (37 per cent)) (*Table S1*). There was no significant difference in the likelihood of missing data between treatment groups (OR 1.21, 95 per cent c.i. 0.80 to 1.83; *P* = 0.372 (*Table S2*)). Data on the key secondary outcomes of perianal fistula and abscess recurrence were available for 100 per cent of participants. These data were collected as SAEs from the participant’s clinical notes, even when the participant was lost to routine trial follow-up.

### Primary outcome: mean maximum pain score over the first 10 postoperative days

**Fig. 2:**
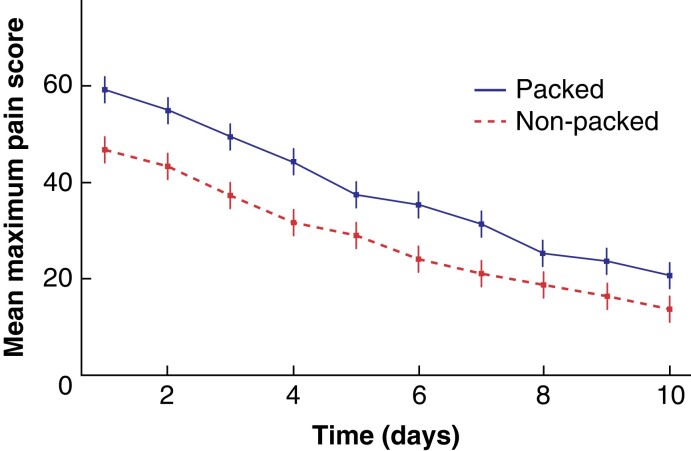
Daily mean maximum pain scores (visual analogue scale) over the first 10 postoperative days

Mean maximum pain over the first 10 postoperative days was significantly lower in the no-packing group: the mean difference was 9.9 (95 per cent c.i. 4.84 to 15.05; *P* < 0.001), which was a 26.2 per cent reduction in pain in the no-packing arm *versus* the packing arm. Pain profiles over the 10 days following the incision and drainage operation are included in *Fig. 2* and *Table S3*.

This pain reduction was consistent under all sensitivity analyses, including a per-protocol analysis, which removed all major protocol deviations, including treatment arm crossovers; the mean maximum pain score difference was 13.7 (95 per cent c.i. 8.5 to 18.9; *P* < 0.001) (*Table 2*). In the packing group 126/213 (59 per cent) of participants had analysable primary outcome data *versus* 145/220 (66 per cent) participants in the no-packing group.

**Table 2:** Primary outcome sensitivity analyses

Sensitivity analysis	Packing group (*n* = 213)		No-packing group (*n* = 220)		Difference in mean maximum pain scores (95% c.i.)	*P*
	No. patients with available data	Mean maximum pain (s.e.)	No. patients with available data	Mean maximum pain (s.e.)		
**Stratified mixed model**	126	38.2 (1.89)	145	28.2 (1.77)	10.8 (5.55–16.06)	<0.001
**Per protocol**	108	38.6 (2.05)	121	24.9 (1.66)	13.7 (8.48–18.89)	<0.001
**Outlier**	126	38.2 (1.89)	144	27.8 (1.72)	10.4 (5.38–15.45)	<0.001
**Excluding patients prescribed metronidazole**	104	38.2 (1.99)	123	28.5 (2.02)	9.7 (4.09– 15.28)	0.001

Stratified model: results from a linear model, including patient site as a main effect. Per protocol: analysis removing 42 patients who had a major protocol deviation (including treatment arm crossover) and provided sufficient data to define the primary endpoint. Outlier: analysis removing patients identified as having outlying data points. Excluding participants prescribed metronidazole: analysis removing 44 patients who received metronidazole (possible confounder) and provided sufficient data for the primary analysis.

Missing primary outcome data was equal between groups. Exploratory analysis on treatment arm, sex, age (less than 40 years, more than 40 years), and smoking status investigated the relationship between these covariants and the probability of observing missing data. No difference was detected between the treatment arms (χ^2^  *P* = 0.176) or sex (*P* = 0.564). Participants aged 40 and over were more likely to return the symptoms diary (less than 40 years of age 41 per cent missing, more than 40 years of age 32 per cent missing; close to statistical significance *P* = 0.051). Current smokers were much more likely to have missing pain diaries (never smoked: 26 per cent missing; current smoker: 50 per cent missing; ex-smoker: 26 per cent missing (*P* < 0.001)). Multiple strategies were employed to minimize loss to follow-up (*Table S2*).

### Secondary outcomes: pain before, during, and after dressing change

Pain scores both during dressing change (mean difference 10.2, 95 per cent c.i. 4.1 to 16.3; *P* < 0.001) and after dressing change (mean difference 5.8, 95 per cent c.i. 0.1 to 11.5; *P* = 0.007) favoured the no-packing group (*Table 3*).

**Table 3:** Secondary outcomes

Outcome	Level	Packing group	No-packing group	*P*
**Pain prior to dressing change**	*n* (%)	104 (49)	113 (51)	
	Mean (s.e.)	30 (1.9)	26 (2)	0.05
**Pain at dressing change**	*n* (%)	104 (49)	112 (51)	
	Mean (s.e.)	42 (2.1)	32 (2)	<0.001
**Pain following dressing**	*n* (%)	104 (49)	113 (51)	
	Mean (s.e.)	32 (1.9)	26 (2.1)	0.007
**Healing rate (at 26 weeks)**	*n*	163	175	
	No	51	37	
	Yes	112	138	0.05
**Metronidazole prescribed**	*n* (%)	202	212	
	No	166 (78)	174 (79)	1
	Yes	36 (17)	38 (17)	
**Patient Global Assessment**	*n* (%)	125	141	
	Poor	6 (5)	1 (1)	
	Fair	29 (23)	20 (14)	
	Good	68 (54)	88 (62)	
	Excellent	22 (18)	32 (23)	0.03
**Patient satisfaction**	*n*	116	134	
	Strongly disagree	3(3)	2 (1)	
	Disagree	6 (5)	0 (0)	
	Neutral	8 (7)	6 (4)	
	Agree	37 (32)	53 (40)	
	Strongly agree	62 (53)	73(54)	
	Median (i.q.r.)	5 (4–5)	5(4–5)	0.06
**Quality of life**	*n*	123	139	
	Day 1	58 (1.90)	58 (1.85)	0.76
	Day 7	73 (1.82)	76 (1.80)	0.19
	Day 14	84 (1.43)	83 (1.80)	0.86
	Day 21	85 (1.75)	86 (1.86)	0.52
	Overall	67 (1.26)	68 (1.27)	0.66

### Secondary endpoints

Fistula was seen by 6 months of follow-up in 32/213 (15 per cent) of the packing group and 24/220 (11 per cent) of the non-packing group (OR 0.69, 95 per cent c.i. 0.39 to 1.22; *P* = 0.204). Despite no detectable statistically significant difference, the OR of 0.69 reflects a likelihood of lower fistula rate in the no-packing group. However, this study was not powered to detect non-inferiority. These data suggest that one perianal fistula would be avoided for every 25 patients not packed (number needed to treat (NNT) 25). Abscess recurrence was low; 7/213 (3 per cent) in the packing group and 13/229 (6 per cent) in the no-packing group, likely favouring the packing group but with wide imprecision around the confidence intervals, and no statistically significant difference detected (OR 1.85, 95 per cent c.i. 0.72 to 4.73; *P* = 0.200). Again, this study was not powered to detect non-inferiority; however, these data suggest that one additional abscess recurrence would occur for every 37 patients not packed (NNT 37).

Further secondary endpoints are included in *Table 3*.

Wound healing (defined as complete epithelialization of the wound) was assessed repeatedly throughout the course of the study and further details are provided in *Table S4*. Over the 6-month follow-up period, a total of 112/163 (69 per cent) in the packing group and 138/175 (79 per cent) in the no-packing group were observed as healed (*P* = 0.046).

The use of metronidazole was similar between groups and was therefore unlikely to impact on the pain data.

Global assessment of care was reported as either good or excellent by 90/125 (72 per cent) and 120/141 (85 per cent) in the packing group and no-packing group, respectively (*P* = 0.033, *Table 3*). With respect to patient satisfaction with wound management: 99/116 (85 per cent) and 126/134 (94 per cent) of participants in the packing group and no-packing group, respectively, agreed or strongly agreed that they were satisfied with the way their wound was managed, and approached statistical significance (*P* = 0.062, *Table 3*).

Details on HRQoL for each component of the EQ-5D^TM^ are included in *Table S5*. Quality of life was assessed on days 1, 7, 14, and 21. Both treatment groups showed a monotonic increase in the mean overall HRQoL. Comparisons between mean scores at each time point are included in *Table S4* and showed no significant differences between the treatment groups.

**Fig. 3:**
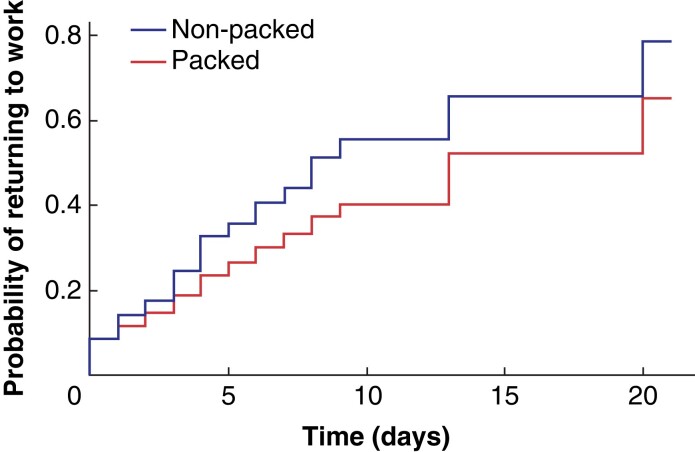
Probability of returning to work over time after incision and drainage operation

Return to work was investigated as a post-hoc secondary outcome. Sixty-five per cent of the packing group *versus* 80 per cent of the non-packing group had returned to work 21 days after their incision and drainage operation (*Fig. 3*).

## Discussion

The PPAC2 trial has demonstrated that postoperative perianal abscess wound cavities managed without internal wound packing result in significantly less pain without significantly increasing adverse events of perianal fistula and abscess recurrence. Global satisfaction with care was greater in the no-packing group and a greater proportion of participants had returned to work by 21 days. The baseline demographics are consistent with those reported in a large population-based observational study^6^. One in six had diabetes mellitus, slightly higher than the 8 per cent reported^1^ in a series with fistula related to inflammatory bowel disease (a lower rate of concomitant diabetes is expected).

Pain in the no-packing group was significantly reduced compared with the packing group, quantified by the primary outcome of mean worst daily pain scores. Daily maximum pain was consistently lower in the non-packing group over the first 10 days. Additionally, pain was significantly higher in the packing group during dressing change, which persisted afterwards. This is consistent with data reported in our observational study^7^. Pain was identified by participants in the PPAC feasibility study, as the most important postoperative outcome^7^, and is now known to be a key measure of patient overall experience and satisfaction with their surgical care^14^.

The minimal clinically important difference (MCID) in pain resulting from the use of an analgesic intervention, pharmaceutical, or adjunct has been reported to be 20 per cent^15,16^ or 10 mm or greater^17,18^ on a VAS score. The PROSPECT working group, highlighting the lack of robust guidance, has designed methodology in order to develop international evidence-based recommendations for procedure-specific perioperative pain management^18^. This group has defined a MCID of 10 mm. The current study reports a difference of 9.9 mm, which can be rounded to 10 mm. The current study has also shown larger relative reductions in postoperative pain compared to other trials investigating pain in proctological surgery. The eTHoS trial compared stapled haemorrhoidopexy with traditional excisional surgery; the stapling procedure was less painful than traditional surgery (TS) with a 13 per cent reduction in worst pain on day 7 (10 mm, TS 7.9, SH 6.9, −1.04 effect size)^19^, a lesser effect size than reported here.

The loss of primary outcome data (although not of fistula or abscess recurrence data) is undeniably high. Despite this, the study has sufficient statistical precision to detect differences between the treatment groups. We believe the attrition and data loss is unlikely to have introduced significant bias and has had minimal impact on the strength of the trial findings. We are confident that this reflects the best possible data collection in this context, and that these data are robust. Strategies recommended by Mercieca-Bebber and colleagues to reduce missing patient-reported outcome data (PRO) included simplifying the PRO data set, using a measure acceptable to participants, reduced data and time points, and postal returns^20^. The loss of data may reflect the young working age population, data-entry fatigue, lack of motivation in absence of pain or other significant symptoms, the use of a pen-and-paper approach rather than electronic data entry, and also the nature of an acute, and possibly embarrassing, condition. Missing PRO data in clinical trials is a widely recognized issue^21–23^.

Perianal fistula was an important secondary outcome for this trial. Incidence of fistula up to 6 months of follow-up was similar between the packing group and the no-packing group. The OR reflects a likelihood of lower fistula rate in the non-packing group; however, this study was not powered to detect non-inferiority. The sample size needed to detect a true difference is 980, which was not achievable within the funding and time constraints of this study. These data suggest that one perianal fistula would be avoided for every 25 patients not packed (NNT 25). If this difference were true, reducing the rate of fistula would be a further important reason to avoid wound packing. Assessment of healing and fistula occurrence was made by individuals blinded to treatment allocation. The abscess recurrence rate was low—although no statistically significant difference between treatment groups was detected. These data suggest a likelihood of a higher rate of abscess recurrence rate in the non-packing group with one additional abscess recurrence for every 37 patients not packed (NNT 37). The sample size needed to detect a true difference is again higher than the funding and time constraints of this study allowed, at 900. Perianal fistula commonly requires further surgery and has greater long-term consequences for patients than an acute abscess recurrence. Arguably, a small increase in abscess recurrence risk would be a reasonable tradeoff for a reduction in fistula rate.

HRQoL was similar between the two groups. ‘Usual activities’ and ‘pain/discomfort’ were the most affected parameters, with 75 per cent of participants reported experiencing severe problems in their ‘usual activities’ at day 1. This dropped dramatically by day 7 and stabilized by day 14, with no further improvement beyond this point. Participants in both groups were generally back to usual activities within 2 weeks. It is now accepted that time to return to work is a key indicator of quality of life^24^. Although there was no identified difference in EQ-5D^TM^ scores between the packing and no-packing groups, a greater proportion of participants in the no-packing group had returned to work at 21 days—a real-life measure of quality of life and functional status.

The main limitations of this trial are the loss of primary outcome data, and non-blinding of participants to their treatment. Non-blinding of participants may have led to bias in patient-reported outcomes. There was a small degree of non-compliance with treatment allocation. This trial was conducted in emergency general surgery units at 50 acute hospitals, including small general hospitals and large academic units, so the trial findings are widely applicable. The study has challenged the dogma of wound packing after incision and drainage of perianal abscess, and found it to be unwarranted.

## Collaborators

A. Watson, M. Johnson, L. Hiller, E. Psarelli, L. Murray, A. Smith, S. Brown, B. Singh, C. Newby, O. Ali, A. Sukha, N. Blencowe, S. Narang, N. Reeves, G. Faulkner, S. Rajamanickam, J. Evans, S. Mangam, M. Harilingham, C. J. Smart, S. J. Ward, M. Bogdan, K. Amin, Z. Al-Khaddar, E. Davies, P. Patel, A. Stearns, I. Shaik, J. Hernon, A. Pal, M. Lewis, J. Barker, A. Gerrard, M. Abdel-Halim, P. Shuttleworth, M. J. Lee, A. B. P. Peckham-Cooper, A. G. Hague, C. Challand, C. Steele, N. Fearnhead, S. Van Laarhoven, R. Brady, F. Shaban, N. Wong, W. Ngu, G. Williams, R. Codd, D. Magowan, K. Leong, G. Williams, A. Torrance, B. Bharathan, N. Pawa, H. Kaur Sekhon, I. Singh, A. Alabi, D. Berry, V. Trompetas, J. L. Hughes, R. Lunevicius, R. Lunevicius, K. Mann, S. Dixon, T. Ingram, T. Gilbert, C. Brooks, G. Madzamba, A. Pullyblank, G. Dovell, L. Newton, N. Carter, P. May-Miller, S. Shaikh, R. Shearer, C.Macleod, C. Parnaby, A. Abdelmabod, L. Titu, T. Majeed, R. Hargest, J. Parker, C. Zabkiewicz, N. Reeves, F. Soliman, G. Gossedge, H. Selvachandran, M. Dilworth, D. Vimalachandran, H. Singh, H. Koh, J. Randall, S. Moug, A. Adeosun, G. Dennison, N. Curtis, N. Smart, S. Duff, M. Rahman, F. Wu.

## Supplementary Material

znac225_Supplementary_Data

## Data Availability

The data is stored at the Liverpool Cancer Trials Unit as per the study's ethical approval, but is not available in a public repository.

## Notes

*Disclosure.* The authors declare no conflict of interest.
