# Supplementary material for: Postoperative Packing of Perianal Abscess Cavities (PPAC2): randomized clinical trial
Source: Br J Surg. 2022 Aug 5;109(10):951–7. doi: 10.1093/bjs/znac225 (PMC10364677; doi:10.1093/bjs/znac225)
Supplement: znac225_Supplementary_Data [file znac225_supplementary_data.zip › Collaborators.docx]

**PPAC2 Collaborators**

**Chief Investigator:**

Prof James Hill (Manchester NHS University Foundation Trust)

**Trial Management Committee and Writing Committee:**

Katy Newton (Manchester NHS University Foundation Trust), Jo Dumville (The University of Manchester), Michelle Briggs (The University of Manchester), Jennifer Law (Northwest Surgical Research Collaborative), Julia Martin (Northwest Surgical Research Collaborative),, Lyndsay Pearce (Salford Royal NHS Foundation Trust), Professor Cliona Kirwan (The University of Manchester), Professor Thomas Pinkney (University Hospitals Birmingham NHS Foundation Trust), Richard Jackson (Northwest Surgical Trials Centre, Liverpool Clinical Trials Centre), Alexander Needham (Northwest Surgical Trials Centre, Liverpool Clinical Trials Centre), Simon Winn (Northwest Surgical Trials Centre, Liverpool Clinical Trials Centre), Professor James Hill (Manchester NHS University Foundation Trust).

**Northwest Surgical Trials Centre, Liverpool Clinical Trials Unit:**

Simon Winn, Haley McCulloch, Richard Jackson, Alexander Needham.

**Independent Data Monitoring Committee:**

Professor Steven Brown, Baljit Singh, Mr Chris Newby (University of Nottingham), Katy Newton (Manchester Univesity NHS Foundation Trust), Alexander Needham (Northwest Surgical Trials Centre, Liverpool Clinical Trials Centre), Simon Winn (Northwest Surgical Trials Centre, Liverpool Clinical Trials Centre), Professor James Hill (Manchester University NHS Foundation Trust)

**Trial Steering Committee:**

Professor Angus Watson (Chair, NHS Highland), Mark Johnson (Leeds Beckett University, Louise Hiller (University of Warwick), Katy Newton (Manchester University NHS Foundation Trust), Simon Winn (Northwest Surgical Trials Centre, Liverpool Clinical Trials Centre), Alexander Needham (Northwest Surgical Trials Centre, Liverpool Clinical Trials Centre), Eftychia Psarelli (Northwest Surgical Trials Centre, Liverpool Clinical Trials Centre), Lindsay Murray (Manchester University NHS Foundation Trust), Alistair Smith (Patient representative), Professor James Hill (Manchester University NHS Foundation Trust).

**Participating centres and investigators (*Principle Investigator at each site):**

Royal Bolton Hospital; Gemma Faulkner*, Shenbaga Rajamanickam, Queen Elizabeth The Queen Mother Hospital (Margate); Jessica Evans*, Sudhakar Mangam, Mohan Harilingham, Macclesfield District General Hospital; Christopher J Smart *, Simon J Ward, Monica Bogdan, Khalid Amin, Ziad Al-Khaddar, Furness General Hospital (Cumbria); Emma Davies*, Panna Patel, Norfolk and Norwich University Hospital; Adam Stearns*, Irshad Shaik, James Hernon, Atanu Pal, Michael Lewis, Blackpool Victoria Hospital; Jonathan Barker*, Adam Gerrard, Mostafa Abdel-Halim, Jennifer Law, Paul Shuttleworth, Queen Elizabeth Hospital Birmingham; Thomas Pinkney*, Sheffield Teaching Hospitals NHS Foundation Trust; Matthew J Lee*, Adam BP Peckham-Cooper, Adam G Hague, Cristopher, Challand, Caroline Steele, Addenbrooke's Hospital (Cambridge); Nicola Fearnhead*, Stijn Van Laarhoven, Royal Victoria Infirmary (Newcastle); Richard Brady*, Fadlo Shaban, Nelson Wong, Wee Ngu (Lena), Royal Gwent Hospital (Newport); Gethin Williams*, Rhodri Codd*, Drew Magowan, University Hospital Coventry; Kai Leong*, Gregory Williams, Birmingham City Hospital; Andrew Torrance*, Nottingham University Hospital NHS Trust Queens Medical Centre; Balamurali Bharathan*, West Middlesex University Hospital (Isleworth); Nikhil Pawa*, Harpeet Kaur Sekhon, Inderjit Singh, Andrew Alabi, Southampton General Hospital; David Berry*, Vasileios Trompetas, University Hospital Aintree; Jane L Hughes*, Raimundas Lunevicius*, Raimundas Lunevicius, Kulbir Mann, Steven Dixon, Tanya Ingram,Tim Gilbert, Carol Brooks,Gladys Madzamba, Southmead Hospital (Bristol); Anne Pullyblank*, George Dovell, Lydia Newton, Queen Alexandra Hospital (Portsmouth); Nick Carter*, Peter May-Miller, Aberdeen Royal Infirmary; Shafaque Shaikh*, Rosalyn Shearer, Campbell Macleod, Craig Parnaby, Areeg Abdelmabod, Arrowe Park Hospital (Wirral); Liviu Titu, Talal Majeed, University Hospital of Wales (Cardiff); Rachel Hargest*, Jody Parker, Catherine Zabkiewicz, Nicola Reeves, Faris Soliman, Warrington Hospital; Gemma Gossedge*, Haran Selvachandran, Heartlands Hospital (Birmingham), Mark Dilworth*, Countess of Chester Hospital; Dale Vimalachandran*, Royal Chesterfield Hospital; Harjeet Singh*, Hoey Koh, Bristol Royal Infirmary; Jonathan Randall*, Royal Alexandra Hospital (Paisley); Professor Susan Moug, Broomfield Hospital (Chelmsford); Abidemi Adeosun, Yeovil District Hospital; Godwin Dennison*, Nathan Curtis, Royal Devon & Exeter Hospital; Neil Smart*, Manchester University NHS Foundation Trust, Professor James Hill*, Sarah Duff*, Katy Newton, Mamun Rahman, Fiona Wu.

**Regional Trainee leads:**

Jennifer Law, Oroog Ali, George Ramsay, Anisha Sukha, Natalie Blencowe Sunil Narang, Nicola Reeves.
