# Supplementary material for: Postoperative Packing of Perianal Abscess Cavities (PPAC2): randomized clinical trial
Source: Br J Surg. 2022 Aug 5;109(10):951–7. doi: 10.1093/bjs/znac225 (PMC10364677; doi:10.1093/bjs/znac225)
Supplement: znac225_Supplementary_Data [file znac225_supplementary_data.zip › Supplementary_material.docx]

## Supplementary Material

### Table S1: Patterns of missingness

| **Covariate** | **Level** | **0 (missing)** | **1   (not missing)** | **Total** | **p-value** |
| --- | --- | --- | --- | --- | --- |
| Total |  | 162 | 271 | 433 |  |
| ASA Grade | 1 | 76 (38%) | 125 (62%) | 201 |  |
| ASA Grade | 2 | 62 (36%) | 110 (64%) | 172 |  |
| ASA Grade | 3 | 13 (32%) | 27 (68%) | 40 | 0.803 |
| Intra-op Antibiotic | No | 99 (40%) | 147 (60%) | 246 |  |
| Intra-op Antibiotic | Yes | 55 (32%) | 118 (68%) | 173 | 0.096 |
| TTO Metronidazole | No | 123 (36%) | 217 (64%) | 340 |  |
| TTO metronidazole | Yes | 30 (41%) | 44 (59%) | 74 | 0.567 |
| Number of Intra-op Packs | 1 | 110 (35%) | 200 (65%) | 310 |  |
| Number of Intra-op Packs | 2 | 20 (37%) | 34 (63%) | 54 |  |
| Number of Intra-op Packs | 3 | 5 (38%) | 8 (62%) | 13 |  |
| Number of Intra-op Packs | 4 | 1 (50%) | 1 (50%) | 2 |  |
| Number of Intra-op Packs | 8 | 0 (0%) | 1 (100%) | 1 | 0.976 |
| Height | median (IQR) | 176.5 (168, 181) | 173 (165.775, 180) | 173 (166, 180) | 0.116 |
| Weight | median (IQR) | 82 (67.975, 96.05) | 83.5 (70, 96.3) | 83 (69.75, 96.2) | 0.743 |
| Diabetes | median (IQR) | 0 (0, 0) | 0 (0, 0) | 0 (0, 0) | 0.543 |
| smoker | No | 40 (27%) | 110 (73%) | 150 |  |
| smoker | Yes | 91 (51%) | 87 (49%) | 178 |  |
| smoker | 2 | 25 (26%) | 72 (74%) | 97 | <0.001 |
| Sex | female | 50 (35%) | 94 (65%) | 144 |  |
| Sex | male | 112 (39%) | 177 (61%) | 289 | 0.477 |
| age | median (IQR) | 37 (29, 49) | 42 (32, 54) | 40 (31, 52) | 0 |
| Treatment Arm | Packing | 87 (41%) | 126 (59%) | 213 |  |
| Treatment Arm | Non-Packing | 75 (34%) | 145 (66%) | 220 | 0.176 |

### Table S2: Multivariable analysis for the pattern of missingness

|  | est (se) | OR (95% CI) |
| --- | --- | --- |
| (Intercept) | -0.08 (0.362) | 0.93 (0.456, 1.884) |
| Smoker Current Vs No | -1.12 (0.243) | 0.33 (0.202, 0.524) |
| Smoker Past Vs No | -0.12 (0.305) | 0.89 (0.489, 1.615) |
| age | 0.03 (0.008) | 1.03 (1.01, 1.042) |
| RANDArm (Packing Vs Non-Packing) | 0.19 (0.211) | 1.21 (0.798, 1.828) |

### Table S3: Daily mean maximum VAS pain scores

| **Day** | **Packing Group (N=213)** | | **Non-Packing Group (N=220)** | |
| --- | --- | --- | --- | --- |
|  | **No. pts available data** | **Mean maximum pain (SE)** | **No. pts available data** | **Mean maximum pain (SE)** |
| ***1*** | 124 | 59.0(2.6) | 133 | 46.8(2.4) |
| ***2*** | 123 | 54.9(2.4) | 141 | 43.3(2.3) |
| ***3*** | 125 | 49.5(2.4) | 141 | 37.1(2.3) |
| ***4*** | 121 | 44.2(2.4) | 142 | 31.8(2.1) |
| ***5*** | 123 | 37.4(2.3) | 140 | 29.1(2.1) |
| ***6*** | 123 | 35.3(2.3) | 141 | 24.0(1.9) |
| ***7*** | 124 | 31.4(2.2) | 142 | 20.8(1.9) |
| ***8*** | 124 | 25.2(2.0) | 141 | 18.5(1.8) |
| ***9*** | 121 | 23.7(2.1) | 141 | 16.2(1.8) |
| ***10*** | 122 | 20.6(1.9) | 137 | 13.6(1.7) |

### Table S4: Rate of wound healing

Healing, defined as complete epithelialization of the wound, was recorded at; four, eight and 26 weeks. Please note that if a patient was healed at week 4 they were not followed up at week 8. All patients were approached for their follow-up at week 26. Participants in each group experienced similar healing rates non-packing vs packing: week four 60% vs 59%, week eight 72% vs 55%, week 26 76% vs 71%.

| **Time point** | **Healing Status** | **Packing Group (N=213)** | **Non-Packing Group (N=220)** | **Overall (N=433)** | **P-value** |
| --- | --- | --- | --- | --- | --- |
| ***Week 4:*** | ***Total Attendees*** *(% randomised)* | 125 (59%) | 148 (67%) | 273 (63%) | - |
|  | ***Healed*** *(% Total attendees)* | 74 (59%) | 89 (60%) | 163 (60%) | 0.875 |
|  | ***Not Healed*** *(% Total attendees)* | 51 (41%) | 59 (40%) | 110 (40%) |  |
| ***Week 8:*** | ***Total Attendees*** *(% randomised)* | 47 (22%) | 54 (25%) | 101 (23%) | - |
|  | ***Healed*** *(% Total attendees)* | 26 (55%) | 39 (72%) | 65 (64%) | 0.077 |
|  | ***Not Healed*** *(% Total attendees)* | 21 (45%) | 15 (28%) | 36 (36%) |  |
| ***Week 26:*** | ***Total Attendees*** *(% randomised)* | 94 (44%) | 99 (45%) | 193 (45%) | - |
|  | ***Healed*** *(% Total attendees)* | 67 (71%) | 75 (76%) | 142 (74%) | 0.480 |
|  | ***Not Healed*** *(% Total attendees)* | 27 (29%) | 24 (24%) | 51 (26%) |  |

### Table S5: Health related Quality of life

Health related quality of life (EQ-5D) was recorded in the symptom diary at day 1, 7, 14 and 21. Table 9 shows the distribution of participants’ EQ-5D scores, dichotomised into ‘problems’ and ‘no problems’ for each dimension. ‘No problems’ is defined by a reported score 0 and ‘ problems’ is defined by scores 1 – 4 representing: ‘no problems’, ‘slight problems’, ‘moderate problems’, ‘severe problems’ and ‘extreme problems’ respectively.

| **Dimension** | **Rating** | **Day** | | | | | | | | |
| --- | --- | --- | --- | --- | --- | --- | --- | --- | --- | --- |
|  |  | **Packing Group (N=213)** | | | |  | **Non-Packing Group (N=220)** | | | |
|  |  | **Day 1** (123 pts) | **Day 7** (122 pts) | **Day 14** (120 pts) | **Day 21** (116 pts) |  | **Day 1** (133 pts) | **Day 7** (139 pts) | **Day 14** (133 pts) | **Day 21** (131 pts) |
| ***Mobility*** | ***problems*** | 102 (83%) | 73 (60%) | 34 (28%) | 26 (22%) |  | 101 (76%) | 61 (44%) | 35 (26%) | 19 (15%) |
|  | ***no problems*** | 21 (17%) | 49 (40%) | 86 (72%) | 90 (78%) |  | 32 (24%) | 78 (56%) | 98 (74%) | 112 (85%) |
|  | ***median (IQR)*** | 1(1-2) | 1(0-1) | 0(0-1) | 0(0-0) |  | 1(1-2) | 0(0-1) | 0(0-1) | 0(0-0) |
| ***Self-Care*** | ***problems*** | 83 (67%) | 64 (52%) | 35 (29%) | 23 (20%) |  | 87 (65%) | 57 (41%) | 28 (21%) | 18 (14%) |
|  | ***no problems*** | 40 (33%) | 58 (48%) | 85 (71%) | 93 (80%) |  | 46 (35%) | 82 (59%) | 105 (79%) | 113 (86%) |
|  | ***median (IQR)*** | 1(0-2) | 1(0-1) | 0(0-1) | 0(0-0) |  | 1(0-2) | 0(0-1) | 0(0-0) | 0(0-0) |
| ***Usual Activities*** | ***problems*** | 108 (88%) | 87 (71%) | 56 (47%) | 39 (34%) |  | 118 (89%) | 89 (64%) | 61 (46%) | 38 (29%) |
|  | ***no problems*** | 15 (12%) | 35 (29%) | 64 (53%) | 77 (66%) |  | 15 (11%) | 50 (36%) | 72 (54%) | 93 (71%) |
|  | ***median (IQR)*** | 2(1-3) | 1(0-1) | 0(0-1) | 0(0-1) |  | 2(1-3) | 1(0-1) | 0(0-1) | 0(0-1) |
| ***Pain/Discomfort*** | ***problems*** | 120 (98%) | 99 (81%) | 72 (60%) | 61 (53%) |  | 125 (94%) | 99 (71%) | 56 (42%) | 49 (37%) |
|  | ***no problems*** | 3 (2%) | 23 (19%) | 48 (40%) | 55 (47%) |  | 8 (6%) | 40 (29%) | 77 (58%) | 82 (63%) |
|  | ***median (IQR)*** | 2(1-2) | 1(1-1) | 1(0-1) | 1(0-1) |  | 2(1-2) | 1(0-1) | 0(0-1) | 0(0-1) |
| ***Anxiety/Depression*** | ***problems*** | 54 (44%) | 30 (25%) | 19 (16%) | 26 (22%) |  | 59 (44%) | 38 (27%) | 28 (21%) | 20 (15%) |
|  | ***no problems*** | 69 (56%) | 92 (75%) | 101 (84%) | 90 (78%) |  | 74 (56%) | 101 (73%) | 105 (79%) | 111 (85%) |
|  | ***median (IQR)*** | 0(0-1) | 0(0-0) | 0(0-0) | 0(0-0) |  | 0(0-1) | 0(0-1) | 0(0-0) | 0(0-0) |
| ***Overall Health*** | ***Mean (SD)*** | 57.6 (21.8) | 72.1 (20.0) | 83.2 (15.7) | 84.4 (19.0) |  | 59.0 (21.8) | 76.3 (21.3) | 83.7 (20.5) | 87.6 (19.1) |
